# Appetitive vs. Aversive conditioning in humans

**DOI:** 10.3389/fnbeh.2015.00128

**Published:** 2015-05-19

**Authors:** Marta Andreatta, Paul Pauli

**Affiliations:** Department of Psychology (Biological Psychology, Clinical Psychology, and Psychotherapy), University of WürzburgWürzburg, Germany

**Keywords:** classical conditioning, reward, punishment, startle reflex, skin conductance response

## Abstract

In classical conditioning, an initially neutral stimulus (conditioned stimulus, CS) becomes associated with a biologically salient event (unconditioned stimulus, US), which might be pain (aversive conditioning) or food (appetitive conditioning). After a few associations, the CS is able to initiate either defensive or consummatory responses, respectively. Contrary to aversive conditioning, appetitive conditioning is rarely investigated in humans, although its importance for normal and pathological behaviors (e.g., obesity, addiction) is undeniable. The present study intents to translate animal findings on appetitive conditioning to humans using food as an US. Thirty-three participants were investigated between 8 and 10 am without breakfast in order to assure that they felt hungry. During two acquisition phases, one geometrical shape (avCS+) predicted an aversive US (painful electric shock), another shape (appCS+) predicted an appetitive US (chocolate or salty pretzel according to the participants' preference), and a third shape (CS–) predicted neither US. In a extinction phase, these three shapes plus a novel shape (NEW) were presented again without US delivery. Valence and arousal ratings as well as startle and skin conductance (SCR) responses were collected as learning indices. We found successful aversive and appetitive conditioning. On the one hand, the avCS+ was rated as more negative and more arousing than the CS– and induced startle potentiation and enhanced SCR. On the other hand, the appCS+ was rated more positive than the CS– and induced startle attenuation and larger SCR. In summary, we successfully confirmed animal findings in (hungry) humans by demonstrating appetitive learning and normal aversive learning.

## Introduction

Predicting threat and food is of crucial importance for any organism's survival. In classical conditioning (Pavlov, 1927), a cue precedes an aversive event, such as a mild painful electric shock (aversive unconditioned stimulus, US), or an appetitive event, such as a food pellet (appetitive US), several times. Afterwards, this stimulus alone (now labeled CS, CS+) is able to elicit either defensive or consummatory responses, respectively. The former kind of associative learning is called aversive conditioning, while the latter is called appetitive conditioning.

Although the prediction of appetitive events is as important for survival as the prediction of aversive events, appetitive conditioning is remarkably less investigated in animals (Bouton and Peck, 1989; Koch et al., 1996; McDannald et al., 2011, for a review see Martin-Soelch et al., 2007) as well as in humans (Klucken et al., 2009, 2013; Austin and Duka, 2010; Delgado et al., 2011; Levy and Glimcher, 2011). This lack of research might possibly be due to the complexity of the appetitive paradigm compared to the aversive one. For example, food as a primary reinforcer must be delivered when the organism is hungry in order to be rewarding (for a recent review see Dickinson and Balleine, 1994; Clark et al., 2012). In human research, this difficulty has been overcome by using money (Austin and Duka, 2010; Delgado et al., 2011; Levy and Glimcher, 2011) or erotic pictures (Klucken et al., 2009, 2013). However, neuro-imaging studies have pointed out that primary (i.e., snacks or drinks) and secondary (i.e., money) reinforcers activate some common brain regions (e.g., striatum) but also entail distinct patterns of activation (Delgado et al., 2011; Levy and Glimcher, 2011).

To our knowledge, only a few human conditioning studies have examined the effects of primary appetitive reinforcers like odor (Gottfried et al., 2002), water (Kumar et al., 2008), or food (Prévost et al., 2012). In the first study, Gottfried et al. (2002) associated neutral faces (CSs) with either an unpleasant, a pleasant, or a neutral odor. Interestingly, they found greater activation in the orbitofrontal cortex (OFC) and in the ventral striatum in response to the appetitive CS+ vs. the aversive CS+. They concluded that the OFC processes the value of the odor and is involved in transferring the affective value from the olfactory (US) to the visual (CS) system. The (ventro) striatal activation has been interpreted as reflecting the appetitive CR elicited by the appetitive CS+. In the second study, Kumar et al. (2008) invited their participants (healthy controls and patients with major depression) into the laboratory early in the morning and asked them to abstain from drinking during the night in order to ensure that they were thirsty before the scan. Fractal pictures were CSs and 0.1 ml of water the US. Interestingly, among other activations, healthy participants (but not depression patients) showed greater activation in the ventral striatum to the appetitive CS+, suggesting that this stimulus was processed as rewarding. In the third study, Prévost et al. (2012) also presented fractal pictures as CSs and either sweet or salty snacks as US according to the participants' preference. The CS was presented for 6 s and during the last second a food picture was additionally presented. Every time the food picture showed up, the experimenter placed a piece of food in the participants' hands, who were allowed to consume the snack immediately. Unfortunately, the authors did not report the brain activation to the appetitive CS+ during the classical conditioning phase; however, they observed less cardiac deceleration to the rewarded CS+ compared to the non-rewarded CS–, indicating differential conditioning effects.

The lack of studies using the startle response as an index for appetitive conditioning is surprising, especially considering its broad use in aversive conditioning. Startle response is an ancestral and automatic defensive response toward sudden, unexpected and strong aversive events (Koch, 1999). This defensive response is mediated by a relatively simple neuronal pathway involving the cochlear root neurons, the caudal pontine nucleus of the reticular formation (PnC) and spinal motoneurons (Fendt and Fanselow, 1999; Koch, 1999). Animal studies have revealed that startle potentiation depends on the projections from the amygdala to the PnC (Fendt and Fanselow, 1999; Koch, 1999), whereas startle attenuation depends on an intact nucleus accumbens (NAcc, Koch, 1999). Such modulation of the startle response is a useful implicit measure for the valence of the presented foreground stimuli. Thus, potentiation indicates negative valence, while attenuation indicates positive valence, both without being greatly influenced by cognitive processes (Hamm and Weike, 2005; Andreatta et al., 2010). To our knowledge, only one animal study has investigated appetitive conditioning by measuring startle responses as a dependent measure. Interestingly, the examined rats showed startle attenuation to a CS+ (i.e., light) associated with the delivery of a sucrose solution. Notably, such attenuation was impaired in those animals with NAcc lesions, but not in those with amygdala lesions, suggesting that the NAcc plays a specific role in eliciting appetitive CRs and in attenuating startle responses (Koch et al., 1996).

The current study is to our knowledge the first to translate this appetitive conditioning paradigm to humans by using primary reinforcers as US, i.e., sweet (chocolate Smarties®) or salty (small salty pretzel) food, and startle modulation as a measure of CRs. We expected the appCS+ to trigger strong appetitive CRs as reflected in startle attenuation, enhanced SCR, and positive valence rating as compared to the other stimuli, i.e., the avCS+, and the CS–.

## Materials and methods

### Participants

Forty-two volunteers accepted to participate in the study and received course credits. Nine participants were excluded from the analysis, two because of technical problems, three because they were coded as non-responders (mean startle amplitude < 5 μV), and four because they did not exhibit enough startle responses per condition (minimum = 2; for details, see Materials and Method). In the end, 33 participants were considered in the analysis (16 males; mean age: 22.09 years, *SD*: 2.84; range: 18–29 years). Four participants were not native Germans, and six were left-handed. One participant remained unaware of the CSs-USs associations throughout the experiment (see Procedure), but we decided not to exclude this participant because his responses were normal and did not affect results.

### Materials

#### Unconditioned stimuli (US)

Two kinds of US were used. As aversive US, we applied a mildly painful electric shock on the non-dominant forearm of participants. The electric shock was delivered by means of two electrodes with 9 mm diameter and spacing 30 cm. The electric stimulus consisted of a pulse stimulus with a frequency of 50 Hz and duration of 200 ms, generated by a current stimulator (Digitimer DS7A, Digitimer Ltd, Welwyn Garden City, UK, 400 V, maximum of 9.99 mA). The intensity of the electric shock was determined individually through a threshold procedure described previously (Andreatta et al., 2010). Briefly, participants underwent two series of ascending and descending intensities in intervals of 0.5 mA. They had to rate each stimulus on a visual scale ranging from 0 (feeling nothing at all) to 10 (really intense pain) with 4 as an anchor for the threshold (just noticeable pain). The mean intensity of the electric stimulus was 2.12 mA (*SD* = 0.56) and it was rated as painful (*M* = 6.45, *SD* = 1.73). The appetitive US consisted of either a chocolate (Smarties®) or small salty pretzel. The choice of the appetitive US depended on the participant's individual preference as reported during the preliminary interview. Namely, participants had to report whether they normally eat salty or sweet food during breakfast. They could also freely choose if they preferred the chocolate or the salty pretzel during the experiment itself. In the end, 22 participants chose the chocolate and 11 the small salty pretzel.

#### Conditioned stimuli (CS)

Geometrical shapes (blue square, yellow circle, green triangle, red hexagon) with a diagonal of 8 cm were presented as CSs. The shapes were presented in the middle of a black computer screen for 8 s. One shape (avCS+) was always associated with the aversive US (painful electric shock), one shape (appCS+) was always associated with the appetitive US (chocolate or salty pretzel), one shape (CS–) was never associated with either the aversive or the appetitive USs, and the fourth shape (NEW) was presented in the extinction phase but not during the acquisition phase in order to assure its neutrality.

#### Startle probe

White noise of 103 dB with duration of 50 ms was used as a startle probe. The acoustic stimuli were presented binaurally over headphones and occurred randomly 4–6 s after the shape's onset.

#### Questionnaires

Before and after the experiment, participants had to fill in the German versions of the State-Trait Anxiety Inventory (STAI, Laux et al., 1981) and the Positive Negative Affect Schedule (PANAS, Krohne et al., 1996). The STAI is an inventory to assess participants' trait and/or state anxiety and consists of 20 items for both the trait and the state versions. Participants' anxiety level before (*M* = 37.06, *SD* = 7.80) and after (*M* = 39.33, *SD* = 9.16) the experiment did not change significantly [*t*_(32)_ = 1.61, *p* = 0.117]. Trait anxiety scores in the current sample ranged between 20 and 58 (*M* = 36.6, *SD* = 8.98), which is comparable to the published normal range of adults (Laux et al., 1981). The PANAS (Krohne et al., 1996) is an index for positive and the negative mood. Individuals with high scores on the positive affects scale (PAS) are prone to emotions such as enthusiasm, while individuals with high scores on the negative affects scale (NAS) are prone to emotions such as distress. Each item consists of an adjective, and participants indicate on a scale ranging from 1 (very slightly) to 5 (extremely) to what extent the adjective reflects their feelings at that particular moment. No significant differences were found in participants' negative affect as a result of the experiment [begin: *M* = 11.67, *SD* = 2.29; end: *M* = 12.88, *SD* = 4.69; *t*_(32)_ = 1.55, *p* = 0.130]. Somehow, participants changed significantly their positive mood from the beginning (*M* = 26.72, *SD* = 4.70) to the end (*M* = 23.66, *SD* = 7.02) of the experiment [*t*_(31)_ = 3.11, *p* = 0.004]. This decrease in the participants' positive mood might have been related to the unpleasantness of the paradigm (painful electric shocks as well as aversive white noise were presented).

### Procedure

Upon arrival in the laboratory, participants read and signed an informed consent form approved by the ethics committee of the Department of Psychology of the University of Würzburg. They were not informed about the contingency between CSs and USs. After having filled in the questionnaires, the electrodes were attached and the pain threshold procedure was performed as described above.

During the ***habituation phase***, the four geometrical shapes were presented twice with an inter-trial interval (ITI) varying between 18 and 25 s (mean: 21.5 s). No US or startle probes were delivered during this phase.

Before the acquisition phase, seven bouts of white noise were delivered every 7–15 s in order to decrease the initial startle reactivity.

**Figure 1:**
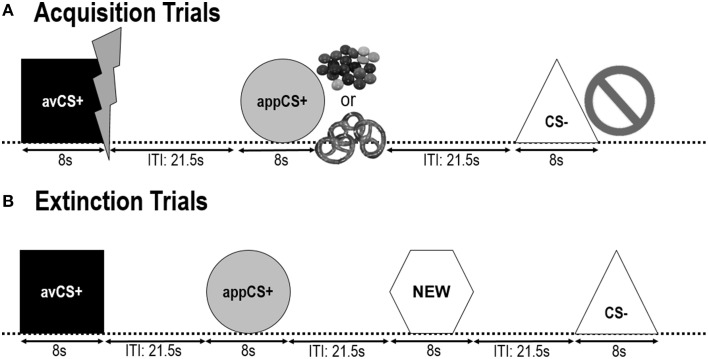
**Trials during the two acquisition phases (A) and the extinction phase (B)**. Participants learned that one shape (avCS+) predicted a mild painful electric shock, one shape (appCS+) predicted either a piece of chocolate or a small salty pretzel (according to their preference), and a third shape (CS–) did not predict any biologically salient event. Each shape was presented in conjunction with a picture depicting an electric shock, smarties/salty pretzel, or nothing depending on the association with the US. During the extinction phase, the three geometrical shapes were presented once again, but no USs were delivered. In addition, a fourth geometrical shape (NEW) was presented as a neutral control.

The following two ***acquisition phases*** were identical (Figure 1). Each acquisition phase consisted of 24 trials: 8 CS– trials, 8 avCS+ trials, and 8 appCS+ trials. The CS sequence was pseudorandom with the only restriction being that the same stimulus could not be presented more than twice in a row. Notably, the avCS+ was presented together with a lightning bolt as a symbol for the electric shock and the painful US was delivered at the offset. The appCS+ was presented in compound with an image of Smarties or a salty pretzel and the participant could pick a Smarties or a pretzel from a jar. The CS– was presented together with a ban symbol and no US was delivered. During three of the 8 CS presentations of each type, a startle probe was delivered between 4 and 6 s after stimulus onset. Three additional startle probes were presented during the ITIs in order to assure their unpredictability and to reduce startle habituation. The ITI, consisting of a black screen, varied between 18 and 25 s with a mean of 21.5 s.

During the ***extinction phase***, participants saw the three geometrical shapes (i.e., the avCS+, the appCS+, and the CS–) again plus a novel neutral shape (NEW). No US was delivered and the shapes were not presented in conjunction with pictures of flashes, chocolate/salty pretzels, or bans. Each stimulus was presented eight times in a pseudorandom order (i.e., the same stimulus was not presented more than twice in a row), creating 32 trials. Startle probe stimuli were presented during 4 out of 8 stimulus presentations of each CS type. As in the acquisition and the habituation phase, the ITIs varied between 18 and 25 s, and 4 additional startle probes were delivered unpredictably during the ITIs.

After each phase, participants rated the valence (pleasantness) and the arousal (excitatory) of the CSs with visual analog scales (VAS) ranging from 1 to 9. The valance scale ranged from “1” indicating “very unpleasant” to “9” indicating “very pleasant;” the arousal scale ranged from “1” indicating “calm” to “9” indicating “exciting.” In addition, contingency ratings were assessed after the two acquisition phases and the extinction phase. Participants saw a geometrical shape for 1 s and then they had to indicate whether this shape was associated with the electric shock, with the chocolate/salty pretzel, with nothing, or whether they were not able to make any association. Notably, all participants (except one) were aware of contingency after Acquisition 2.

### Data reduction

Physiological responses were recorded with a V-Amp 16 amplifier and Vision Recorder V-Amp Edition Software (Version 1.03.0004, BrainProducts Inc., Munich, Germany). A sampling rate of 1000 Hz and a 50 Hz notch filter were applied. The offline analyses were conducted with Brain Vision Analyzer (Version 2.0; BrainProducts Inc., Munich, Germany).

#### Startle response

Startle response was measured by means of electromyography (EMG) at the left *orbicularis oculi* muscle with two 5 mm Ag/AgCl electrodes. In accordance with guidelines (Blumenthal et al., 2005), one electrode was positioned under the pupil and the second one 1 cm laterally. The ground and reference electrodes were placed on the right and left mastoids, respectively. Before attaching the electrodes, the skin was lightly abraded and cleaned with alcohol in order to keep impedance below 10 kΩ. The electromyographic signal was offline filtered with a 28 Hz low cutoff filter and a 500 Hz high cutoff filter. Then, the EMG signal was rectified and a moving average of 50 ms was applied. We used the 50 ms before startle probe onset as a baseline (Grillon et al., 2006). Responses to startle probes were scored manually, and trials with excessive baseline shifts (±5 μV) or movement artifacts were excluded from further analysis. Startle responses lower than 5 μV were coded as zero and considered for the calculation of startle magnitude (Blumenthal et al., 2005). Altogether, 10.4% of trials were rejected, and a minimum of 2 out of 3 startle responses in the acquisition phases and 4 out of 8 startle responses in the extinction phase for each condition were required to keep the participant in the analysis pool. For this reason, four participants were excluded. The peak amplitude was defined as the maximum peak relative to baseline during the 20–120 ms time window after startle probe onset. The raw data were then normalized within-subjects using *z*-scores and then T-scores in order to reduce the influence of individual variability and to better detect psychological processes. The T-scores were averaged for each condition (avCS+, appCS+, CS–, NEW, and ITI). In order to investigate startle potentiation or startle attenuation, the scores for the ITI startle responses were subtracted from the startle responses for each condition.

#### Skin conductance response (SCR)

Skin conductance response (SCR) was recorded using two 5 mm Ag/AgCl electrodes placed on the palm of the non-dominant hand. The galvanic response was offline filtered with a 1 Hz high cutoff filter. The SCR was defined as the difference (in μS) between the response onset (1–3 s after stimulus onset) and the response peak (Tranel and Damasio, 1994; Delgado et al., 2011). Trials containing startle probes were not considered in the analysis of the SCR. Responses below 0.02 μS were coded as zero. Five further participants were excluded from the SCR analysis because they had a mean SCR lower than 0.02 μS. The raw skin conductance data were square root transformed in order to normalize the distribution and scores were averaged for each condition separately for the two acquisition phases (avCS+, appCS+, CS–) and the extinction phase (avCS+, appCS+, CS–, and NEW).

### Statistical analysis

All data were analyzed with SPSS for Windows (Version 20.0, SPSS Inc.). For the physiological responses, separated multivariate analyses of variance (ANOVA) were calculated for the two acquisition phases and the extinction phase. The ANOVA for the acquisition phases had stimulus (avCS+, appCS+, CS–) and phase (Acquisition 1, Acquisition 2) as within-subjects factors. The ANOVA for the extinction phase had only stimulus (avCS+, appCS+, CS–, NEW) as a within-subjects factor. Valence, arousal, and contingency ratings were analyzed with separate ANOVAs containing the within-subjects factors stimulus (avCS+, appCS+, CS–, and NEW) and phase. This factor had four levels for the valence and arousal ratings (T1: after habituation phase, T2: after first acquisition phase, T3: after second acquisition phase, T4: after extinction phase), but three levels for the contingency ratings (T1: after first acquisition phase, T2: after second acquisition phase, T3: after extinction phase).

The alpha (α) level was set at 0.05 for all analyses. The effect size is reported as partial η^2^.

## Results

**Figure 2:**
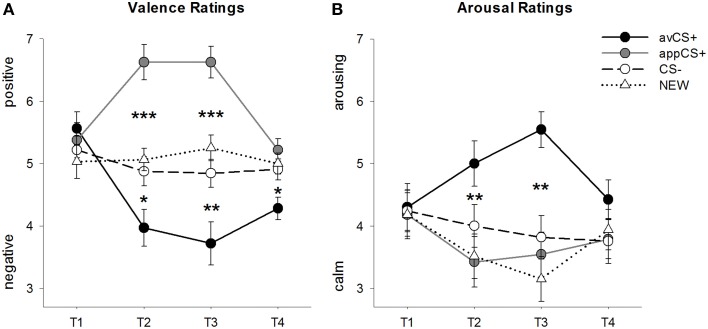
**Ratings for valence (A) and arousal (B)**. Lines (with standard errors) depict the ratings after the habituation phase (T1), Acquisition 1 (T2), Acquisition 2 (T3), and the extinction phase (T4). The aversive CS+ (black solid line) acquired negative valence and high arousal after the two acquisition phases compared to the CS– (black dashed line) and the NEW (black dotted line). Importantly, the appetitive CS+ (gray solid line) acquired positive valence compared to the CS– and the NEW. ^*^p < 0.05, ^**^p > 0.01, ^***^p < 0.001.

**Figure 3:**
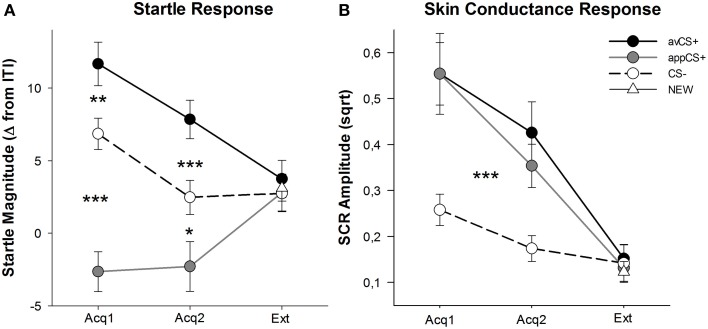
**Startle responses (A) and skin conductance (B) (with standard errors) during the first acquisition phase (Acq1), the second acquisition phase (Acq2), and the extinction phase (Ext)**. Startle responses were significantly potentiated to the aversive CS+ (black solid line) and significantly attenuated to the appetitive CS+ (gray solid line) as compared to the CS– (black dashed line) during the acquisition phases. SCR was significantly greater to the avCS+ and the appCS+ compared to the CS–. No differences were revealed during the extinction phase. ^*^p < 0.05, ^**^p > 0.01, ^***^p < 0.001.

The valence and arousal ratings for each phase are depicted in Figure 2; the startle responses and SCRs are depicted in Figure 3.

### Ratings

The ANOVA for the ***valence ratings*** during acquisition revealed significant main effects of stimulus [*F*_(3, 93)_ = 17.26, GG-ε = 0.801, *p* < 0.001, partial η^2^ = 0.358] and phase [*F*_(3, 93)_ = 3.30, GG-ε = 0.731, *p* = 0.039, partial η^2^ = 0.096] as well as a significant interaction between stimulus and phase [*F*_(9, 279)_ = 9.54, GG-ε = 0.463, *p* < 0.001, partial η^2^ = 0.235]. Follow-up *t*-tests indicate that the valences of the four geometrical shapes at the beginning of the experiment were identical (*p*s > 0.19), while after both Acquisition 1 and Acquisition 2 the avCS+ was rated as especially negative and the appCS+ as especially positive. Specifically, the avCS+ was rated as more negatively valenced compared to the CS– [Acq1: *t*_(31)_ = 2.34, *p* = 0.026; Acq2: *t*_(31)_ = 3.07, *p* = 0.004], the NEW [Acq1: *t*_(31)_ = 2.70, *p* = 0.011; Acq2: *t*_(31)_ = 3.89, *p* < 0.001], and the appCS+ [Acq1: *t*_(31)_ = 5.41, *p* < 0.001; Acq2: *t*_(31)_ = 6.11, *p* < 0.001]. The appCS+ was rated as significantly more positive than the CS– [Acq1: *t*_(31)_ = 4.99, *p* < 0.001; Acq2: *t*_(31)_ = 5.31, *p* < 0.001] and the NEW [Acq1: *t*_(31)_ = 4.92, *p* < 0.001; Acq2: *t*_(31)_ = 4.14, *p* < 0.001]. Differences between the CS– and the NEW were never significant (*p*s > 0.18).

Importantly, no significant differences were found when comparing the valence ratings for the appCS+ associated with chocolate vs. salty pretzel [Acq1: *t*_(30)_ = 0.03, *p* = 0.477; Acq2: *t*_(30)_ = 0.29, *p* = 0.775].

After the extinction phase, the avCS+ was still rated as more negative compared to the CS– [*t*_(31)_ = 2.40, *p* = 0.023], the NEW [*t*_(31)_ = 3.40, *p* = 0.002], and the appCS+ [*t*_(31)_ = 3.35, *p* = 0.002]. On the contrary, the valence of the appCS+ did not differ any more from the CS– [*t*_(31)_ = 1.77, *p* = 0.086] or the NEW [*t*_(31)_ = 1.07, *p* = 0.293].

The ANOVA for the ***arousal ratings*** during acquisition revealed a significant main effect of stimulus [*F*_(3, 96)_ = 7.07, GG-ε = 0.737, *p* = 0.001, partial η^2^ = 0.181], but not of phase [*F*_(3, 96)_ = 1.27, GG-ε = 0.805, *p* = 0.289, partial η^2^ = 0.038], and a significant interaction between stimulus and phase [*F*_(9, 288)_ = 4.53, GG-ε = 0.582, *p* = 0.001, partial η^2^ = 0.124]. Follow-up *t*-tests indicated no significant differences among the stimuli regarding their initial arousal (*p*s > 0.74). However, after the two acquisition phases, the avCS+ was rated more arousing than the NEW [Acq1: *t*_(32)_ = 2.99, *p* = 0.005; Acq2: *t*_(32)_ = 5.97, *p* < 0.001] and the appCS+ [Acq1: *t*_(32)_ = 2.62, *p* = 0.013; Acq2: *t*_(32)_ = 4.42, *p* < 0.001], and marginally more arousing than the CS– after the first acquisition phase [*t*_(32)_ = 1.96, *p* = 0.058], but significantly more arousing after the second acquisition phase [*t*_(32)_ = 3.65, *p* = 0.001]. In contrast to the valence ratings, arousal of the appCS+ did not differ from the arousal of the CS– and the NEW (*ps* > 0.13) after the acquisition phases.

Similarly as with the valence ratings, CS– and NEW did not differ in their arousal (*p*s > 0.07).

As was the case for valence, no differences were revealed for the appCS+ associated with the chocolate and the appCS+ associated with the salty pretzel [Acq1: *t*_(31)_ = 0.26, *p* = 0.797; Acq2: *t*_(31)_ = 0.33, *p* = 0.724].

After extinction phase, no significant differences in the arousal ratings were revealed (*p*s > 0.08).

For additional *post-hoc t*-tests comparing the ratings after the habituation phase, the first and the second acquisition phases and the extinction phase, see Supplemental Materials.

### Startle response

The ANOVA for the acquisition phases returned a main effect of stimulus [*F*_(2, 64)_ = 49.92, GG-ε = 0.964, *p* < 0.001, partial η^2^ = 0.609], but not phase [*F*_(1, 32)_ = 3.16, *p* = 0.085, partial η^2^ = 0.090], and a significant interaction Stimulus × Phase [*F*_(2, 64)_ = 3.37, GG-ε = 0.875, *p* = 0.048, partial η^2^ = 0.095]. Follow-up *t*-tests revealed significant startle potentiation to the avCS+ compared to the CS– during both the first [*t*_(32)_ = 3.27, *p* = 0.003] and the second [*t*_(32)_ = 4.00, *p* < 0.001] acquisition phases. Startle responses to the avCS+ were also significantly potentiated compared to those to the appCS+, again after both Acquisition 1 [*t*_(32)_ = 8.20, *p* < 0.001] and Acquisition 2 [*t*_(32)_ = 5.74, *p* < 0.001]. Importantly, startle magnitude to the appCS+ was significantly attenuated compared to the CS– during both Acquisition 1 [*t*_(32)_ = 6.34, *p* < 0.001] and Acquisition 2 [*t*_(32)_ = 2.91, *p* = 0.007]. Again and in line with the ratings, no differential startle responses were revealed for the appCS+ when associated with the chocolate or with the salty pretzel [Acq1: *t*_(31)_ = 1.04, *p* = 0.309; Acq2: *t*_(31)_ = 0.07, *p* = 0.947]. During the extinction phase, no significant effect was found [*F*_(3, 96)_ = 0.26, GG-ε = 0.906, *p* = 0.833, partial η^2^ = 0.008].

### Skin conductance response (SCR)

From the ANOVAs for the SCR during the two acquisition phases, the main effects stimulus [*F*_(2, 54)_ = 18.04, GG-ε = 0.908, *p* < 0.001, partial η^2^ = 0.401] and phase [*F*_(1, 27)_ = 20.91, *p* < 0.001, partial η^2^ = 0.436] turned out to be significant, but not their interaction [*F*_(2, 54)_ = 0.68, GG-ε = 0.637, *p* = 0.451, partial η^2^ = 0.024]. *Post-hoc t*-tests indicated significantly greater SCR to the avCS+ [*t*_(27)_ = 6.46, *p* < 0.001] and to the appCS+ [*t*_(27)_ = 4.84, *p* < 0.001] compared to the CS–, while participants showed comparable SCR to the avCS+ and the appCS+ [*t*_(27)_ = 0.64, *p* = 0.527]. Notably, no differences in SCRs to the chocolate appCS+ and to the salty pretzel appCS+ were found [Acq1: *t*_(26)_ = 2.55, *p* = 0.120; Acq2: *t*_(26)_ = 1.29, *p* = 0.210]. As was true for the ratings and the startle response, no significant effects were found for the extinction phase [*F*_(3, 81)_ = 0.28, GG-ε = 0.634, *p* = 0.743, partial η^2^ = 0.010].

## Discussion

The goal of this study was to translate animal findings to humans by using a classical appetitive conditioning paradigm with a primary reinforcer as unconditioned stimulus (US). In order to do so, participants came to the lab early in the morning without breakfast in order to assure that they were hungry, and according to their preference, pieces of chocolate or salty pretzels were used as appetitive USs. During the acquisition phase, one geometrical shape (avCS+) became associated with a mildly painful electric shock (aversive US), another shape (appCS+) with the appetitive US, and a third shape (CS–) with neither the appetitive US nor with the aversive US. Results indicate successful aversive and appetitive conditioning on the explicit verbal level (i.e., ratings), on the implicit behavioral level (i.e., startle response), and on the physiological level (i.e., SCR). Specifically, the avCS+ compared to the CS– elicited more negative valence ratings, higher arousal ratings, startle potentiation, and greater SCR. Most importantly, the appCS+ compared to the CS– triggered more positive valence ratings, startle attenuation, and greater SCR. Our findings on aversive conditioning were in line with expectations, since previous studies have found that a stimulus predicting threat (avCS+) is rated as aversive, elicits greater fear responses, and increases physiological arousal (Fendt and Fanselow, 1999; Hamm and Weike, 2005; Andreatta et al., 2010, 2013). Our results on appetitive conditioning were also in line with previous human and animal studies showing that a stimulus predicting reward (appCS+) is rated as positive, inhibits fear responses, and increases physiological arousal (Koch et al., 1996; Gottfried et al., 2002; Kumar et al., 2008; Klucken et al., 2009, 2013; Austin and Duka, 2010; Prévost et al., 2012). To our knowledge, this is the first study demonstrating conditioned startle attenuation in humans to a stimulus predicting a primary reward. Importantly, we were able to transfer and confirm the results of an animal study (Koch et al., 1996). This animal study demonstrated that startle attenuation in rats depends on projections from the NAcc (part of the ventral striatum) to the PnC. Therefore, the startle attenuation in our study could imply NAcc activity, which is also in line with fMRI findings (Gottfried et al., 2002; Kumar et al., 2008; Klucken et al., 2009, 2013; Delgado et al., 2011; Levy and Glimcher, 2011). Hence, we conclude that our appetitive conditioning paradigm was successful, as both the explicit (ratings) and the implicit (startle attenuation) positive valence indicated.

In addition to this new but rather predictable finding, two further interesting results deserve mention. First, verbal and physiological arousal responses to the appCS+ dissociated. Second, we found faster extinction of the appetitive CRs compared to the aversive CRs.

The SCR indicates sympathetic activation, which is increased to both the reward-associated stimulus (appCS+) and the threat-associated stimulus (avCS+). This result is in line with a previous conditioning study, in which erotic pictures were used as appetitive US (Klucken et al., 2013), and a study revealing comparable SCR to a cue predicting money and to a cue predicting an aversive noise (Austin and Duka, 2010). Notably, SCR is an orienting response related to the activation of the sympathetic system. This response has been suggested to reflect the preparation of a behavioral reaction to motivationally salient events (Bradley, 2009). Based on this, we think that both the threat-associated and the reward-associated stimuli elicited a preparatory response for successive behavioral responses. In other words, food and pain signals initiated preparation for approach and avoidance behavior, respectively. In contrast to the high physiological arousal, verbal responses indicated low arousal to the reward-associated stimulus. It is possible that verbal arousal might be more influenced by the arousing nature of the US rather than the physiological activation *per se*. In fact, we think that the appCS+ vs. the avCS+ were rated as lowly vs. highly arousing because they were linked to lowly and highly arousing USs, respectively. Unfortunately, we did not collect arousal ratings for the USs, and therefore it will be up to future studies to test this hypothesis explicitly.

During the extinction phase, no US was delivered. This may have induced a new inhibitory learning called extinction (for a review see Milad and Quirk, 2012), with both the aversive and the appetitive CRs decreasing as a consequence. Evidently, on the behavioral (i.e., startle response) and on the physiological (i.e., SCR) levels, no differential reactions to the avCS+, the appCS+, and the CS– were detectable anymore. Similarly, the explicit arousal of the conditioned stimuli assessed after the extinction phase equalized at a low level, suggesting successful extinction learning. However, the avCS+ was still rated significantly more negative than the CS–, whereas the appCS+ valence did not differ from the CS– valence anymore. The slower extinction of the aversive explicit response might be due to an evolutionary conservatism, meaning that threat signals are especially hard to forget because a non-response to a threat signal might be life threatening.

One question still remains: why then did the startle response (i.e., the implicit valence) extinguish completely during the extinction phase? First, these responses were calculated over the entire course of the phase. Therefore, it is conceivable that discriminative CRs would be still detectable during the first trials of the extinction phase. In an explorative manner, we followed this hypothesis and looked at both the startle responses and SCR throughout the extinction phase (see Supplemental Material). Although we did not find significant differences, we observed slightly higher startle magnitude for the avCS+ as compared to the CS– and the novel control stimulus. We also observed slightly more startle attenuation to the appCS+ as compared to the CS– and the novel control stimulus at the very beginning of the extinction phase, which, however, disappeared within a few trials. SCR to the appCS+ dropped already after 2nd extinction trial, while the SCR to the avCS+ remained higher for almost all extinction compared to the SCR to the NEW. Although we should interpret these results with great caution, the startle responses seemed to parallel the valence ratings. Moreover, both startle responses and SCRs further support the idea of an evolutionary conservatism toward threat stimuli.

Finally, we have to acknowledge some limitations of this study. First, due to a technical problem we could not report ratings of pleasantness (and arousal) for the chocolate and the salty pretzel. However, the appetitive CRs suggest that participants indeed experienced the two USs as appetitive. Second, the duration of the aversive and the appetitive US greatly differed. Thus, the painful electric shock was delivered at the offset of the avCS+ and lasted exactly 200 ms, while the chocolate and the salty pretzel were presented to participants circa 2 s after the appCS+ onset, and the duration was undefinable because it depended on how quickly the individual ate them. The choice to deliver the appetitive USs in this way was based on a previous study in humans (Prévost et al., 2012). However, it would be methodologically more elegant to deliver an appetitive US more comparable to the aversive US, such as a sip of juice or ginger ale at appCS+ offset. In order to compensate for this large perceptual difference, we presented geometrical shapes in conjunction with a visual stimulus symbolizing the US. Third, we cannot definitely exclude the possibility that the quickly extinguished physiological responses in the extinction phase are due to a methodological aspect. In fact, the visual stimuli in this phase were not presented in conjunction with the US symbol as was the case in the acquisition phases. It is possible that the presentation of the CSs alone may have influenced the CRs and for that reason no significant differences were visible during the first extinction trials. However, our quick extinguished responses are in line with the extinguished responses in a previous study in which the CS+ (face) was presented in compound with the US (scream) during acquisition but not during extinction (Lissek et al., 2008).

In conclusion, we found successful aversive and appetitive conditioned responses to a stimulus associated with threat and to a stimulus associated with reward, respectively. Interestingly, the explicit (ratings) and the implicit (startle reflex) level of responses worked in a synergic manner, in that the avCS+ was reported as negative and induced startle potentiation and the appCS+ was reported as positive and induced startle attenuation. Furthermore, explicit (ratings) and physiological (SCR) arousal of the appCS+ dissociated, reflecting two distinct processes.

### Conflict of interest statement

The authors declare that the research was conducted in the absence of any commercial or financial relationships that could be construed as a potential conflict of interest.
