# Supplementary material for: Appetitive vs. Aversive conditioning in humans
Source: Front Behav Neurosci. 2015 May 19;9:128. doi: 10.3389/fnbeh.2015.00128 (PMC4436895; doi:10.3389/fnbeh.2015.00128)
Supplement: Supplementary file 1 [file DataSheet1.DOCX]

**Appetitive vs. Aversive Conditioning in Humans**

Marta Andreatta, Paul Pauli

Department of Psychology (Biological Psychology, Clinical Psychology and Psychotherapy),

University of Würzburg, Würzburg, Germany

**Additional post-hoc *t*-tests**

**Post-hoc *t*-tests for the valence ratings throughout the phases.** The avCS+ was rated significantly more negatively after Acquisition 1 compared to its initial ratings (*t*(31) = 4.31, *p* < .001). No difference in avCS+ valence were revealed between the two acquisition phases (*t*(31) = 0.94, *p* = .354) and between Acquisition 2 and the extinction phase (*t*(31) = 1.36, *p* = .184). Thus, the avCS+ acquired and maintained a negative valence.

The appCS+ was rated with significantly more positive valence after Acquisition 1 compared to its initial valence (*t*(31) = 3.69, *p* = .001), and no differences were revealed between the two acquisition phases (*t*(31) < .1). After the extinction phase, the appCS+ was rated as significantly more neutral compared to Acquisition 2 (*t*(31) = 5.02, *p* < .001). Thus, the appCS+ did become more rewarding, but after a few test trials, the appetitive conditioned responses extinguished.

No significant differences were found for the CS- (*p*s > .169) and the NEW (*p*s > .234).

**Post-hoc *t*-tests for the arousal ratings throughout the phases.** Arousal ratings of the avCS+ did not differ significantly between Acquisition 1 and the habituation phase (*t*(32) = 1.63, *p* = .113) and only marginally between Acquisition 1 and Acquisition 2 (*t*(32) = 1.79, *p* = .083). However, the avCS+ was reported with significantly higher arousal after Acquisition 2 than its initial arousal (*t*(32) = 3.63, *p* = .001). After the extinction phase, the avCS+ was rated as significantly less arousing than after Acquisition 2 (*t*(32) = 4.57, *p* < .001). Thus, after the acquisition phases the avCS+ became slightly more arousing, but its arousal decreased after a few trials during which no US was delivered.

The arousal rating of the appCS+ after the first acquisition only marginally increased compared to the initial ratings (*t*(32) = 1.83, *p* = .077). No differences were revealed between the two acquisition phases (*t*(32) = 0.51, *p* = .613) and between the second acquisition and the extinction phase (*t*(32) = 0.74, *p* = .462). Thus, the appCS+ did not become more arousing by means of its association with the appetitive US.

Arousal of the CS- did not change significantly throughout the experiment (*p*s > .360).

Somewhat surprisingly, arousal ratings for the novel control stimulus (NEW) significantly decreased after Acquisition 1 compared to the habituation phase (*t*(32) = 2.92, *p* = .029) and even more after Acquisition 2 compared to Acquisition 1 (*t*(32) = 2.04, *p* = .050). After the extinction phase, the NEW was rated with increased arousal compared to after the Acquisition 2 (*t*(32) = 2.95, *p* = .006), at a level roughly equal to its initial arousal (*t*(32) = 0.88, *p* = .385).

**Additional statistical analysis.** In order to verify whether the aversive and the appetitive memories related to the respective CSs were present at the beginning of the extinction phase, we considered the response to each condition throughout the extinction phase. Notably, each stimulus (avCS+, appCS+, CS- and NEW) was presented eight times during the extinction phase, and during four out of eight stimulus presentations, the startle eliciting stimulus was delivered. Therefore, the first two startle responses (T1 and T2) depict the startle responses during the first half of the extinction phase, while the last two startle responses (T3 and T4) were elicited during the second half of the extinction phase. The remaining four trials without the startle probes were considered for the SCRs.

We then calculated two ANOVAs containing stimulus (avCS+, appCS+, CS-, NEW) and trial (T1, T2, T3, T4) as within-subject factors. The alpha (α) level was set at .05 for all analyses and the effect size reported as partial η^2^.

**Startle responses.** No main or interaction effects turned out to be significant (*p*s > .314).

Descriptively, startle responses were slightly potentiated to the avCS+ compared to the CS- and the NEW during the first half of the extinction phase. During the second half of the extinction phase, these differences were not detectable anymore. With regard to the appCS+, we observed slight startle attenuation, which disappeared after a very small number of trials during the extinction phase, indicating quick extinction processes (see Supplementary Figure 1).

**SCR.** The main effect trial turn out significant (*F*(3, 78) = 7.31, GG-ε = .774, *p* = .001, partial η^2^ = .219) indicating SCR habituation through the trials. No main effect stimulus (*F*(3, 78) = 0.33, GG-ε = .613, *p* = .705, partial η^2^ = .012) or Stimulus x Trial interaction (*F*(9, 234) = 0.48, GG-ε = .633, *p* = .815, partial η^2^ = .018) were significant (see Supplementary Figure 2).


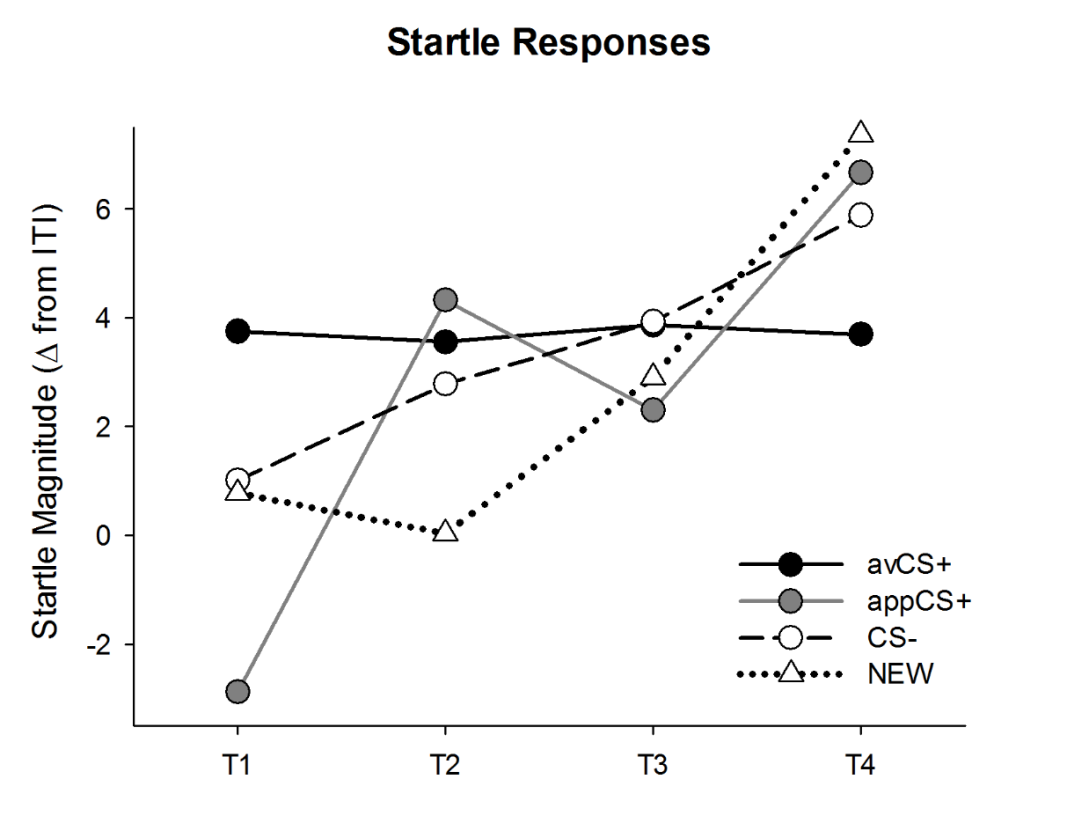


***Supplemental Figure 1*.** Lines (without standard errors, only for display) depict startle responses to the aversive CS+ (black solid line), the appetitive CS+ (grey solid line), the CS- (black dashed line), and the NEW (black dotted line) during the first (T1), the second (T2), the third (T3), and the fourth (T4) delivery of the startle-eliciting stimulus during the extinction phase.


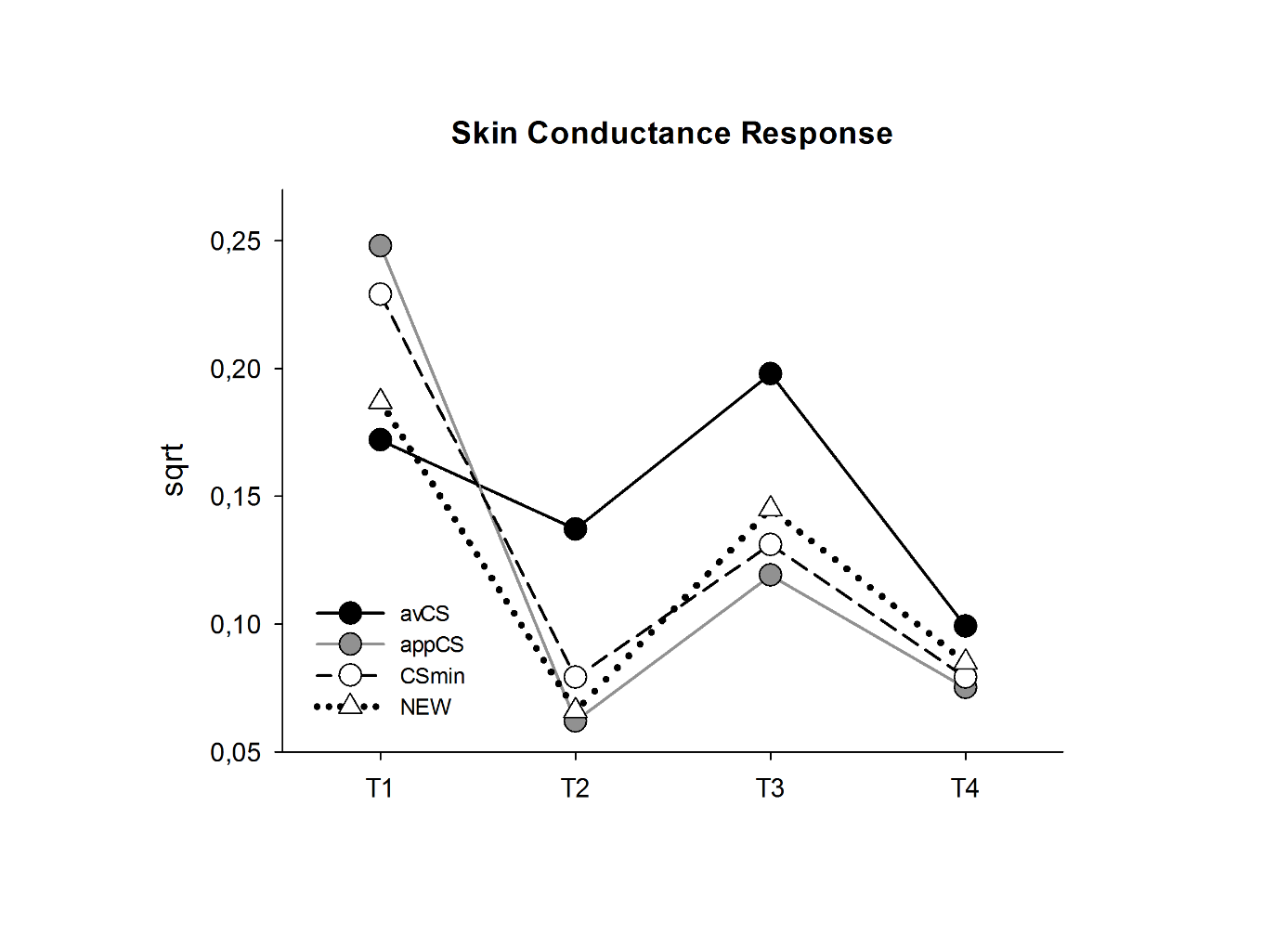


***Supplemental Figure 2*.** Lines (without standard errors, only for display) depict SCR to the aversive CS+ (black solid line), the appetitive CS+ (grey solid line), the CS- (black dashed line), and the NEW (black dotted line) during the first (T1), the second (T2), the third (T3), and the fourth (T4) trial of the extinction phase.
